# Effects of timed LED regimes on tomato plant traits, performance of two‐spotted spider mites, and predatory mites ( *Phytoseiulus persimilis* )

**DOI:** 10.1002/ps.8630

**Published:** 2025-01-09

**Authors:** Patrice Savi, Samantha Hall, Maria Hernandez, Anil Mantri, Daniel Kliebenstein, Christian Nansen

**Affiliations:** ^1^ Department of Entomology and Nematology University of California Davis USA; ^2^ Department of Plant Sciences University of California Davis USA

**Keywords:** timed LED regimes, greenhouse, integrated pest management, spider mites, predatory mites

## Abstract

**BACKGROUND:**

Light‐emitting diodes (LEDs) are being used in controlled environments to enhance crop production and pest management with most studies focusing on continuous treatments (applied throughout the entire daytime or nighttime period). Here, we tested the hypothesis that providing tomato plants with timed LED regimes (daily 3‐h doses of red, blue, or far‐red LED) during the day or at night may affect their traits (leaf reflectance indices, element composition, and phenolic profile), performance of two‐spotted spider mites (*Tetranychus urticae*) (TSSM), and a species of predatory mite (*Phytoseiulus persimilis)*.

**RESULTS:**

Nighttime LED regimes significantly altered leaf element composition: red LED increased K levels, blue LED enhanced Mg levels, and far‐red LED enhanced Mn and Cu and reduced Zn levels. Among daytime LED regimes, blue LED reduced Zn level. Nighttime LED regimes significantly increased leaf glandular trichome densities (except far‐red regime) and reduced total phenolic content. Among a series of leaf reflectance indices, ARI and CRI increased significantly in response to nighttime red and blue regimes but decreased with far‐red LED regimes. Performance bioassays showed significantly lower TSSM populations on nighttime plants than on daytime and control plants. LED regimes did not affect predatory mites' population and their feeding capacity, except for blue daytime regime, which was reduced on mobile TSSM. These results suggest that timed LED regimes have potential to strategically manipulate plant‐prey–predator interactions.

**CONCLUSION:**

We conclude that timed LED regimes can be crucial in designing integrated pest management strategies that promote both plant growth and effective biological control in controlled environments. © 2025 The Author(s). *Pest Management Science* published by John Wiley & Sons Ltd on behalf of Society of Chemical Industry.

## Graphical abstract

Light‐emitting diode (LED)‐based treatments for enhancing crop production and pest management have primarily focused on continuous treatments. This study, using *Tetranychus urticae* and *Phytoseiulus persimilis*, demonstrates that the timing of LED supplementation is crucial for designing integrated pest management strategies that improve both plant growth and effective biological control in controlled environments.

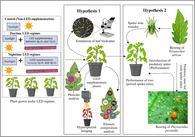

## 1 INTRODUCTION

Due to low and inconsistent availability of natural sunlight, supplementary artificial light sources, particularly light‐emitting diodes (LEDs), are widely integrated into high‐intensity controlled environment crop productions.^1^, ^2^ An important and unique feature of supplementary LEDs is that they can be tailored to specific plant requirements. For instance, deployments of supplementary red and blue LEDs in tomato greenhouse production boosted yields and accelerated ripening by 1 week in spring and 2 weeks in summer, resulting in a 16% increase in cumulated productivity compared to non‐LED treatment.^3^ Potential benefits of deploying supplementary LEDs extend beyond direct yield benefits. Moreover, supplementary LEDs have being examined in efforts to, directly or indirectly, manipulate dynamics of arthropod pest populations and their interactions with natural enemies.^2^, ^4^, ^5^

Direct effects of LEDs on arthropod pests and their natural enemies may arise through phototactic responses. Phototaxis refers to specific behavior in response to light stimuli, which can be either positive (i.e., moving toward a light source) or negative (i.e., moving away from light). Specific phototactic responses of arthropod pests and natural enemies can vary depending on light quality, intensity.^6^, ^7^, ^8^ Phototactic responses have been used as innovative approaches to improve pest management strategies.^7^, ^9^, ^10^ For instance, positive phototactic responses have been used to trap pest species and/or attract natural enemies.^11^, ^12^, ^13^, ^14^, ^15^, ^16^, ^17^, ^18^ Conversely, negative phototactic responses have been used as deterrent to minimize immigration of arthropod pests into cropping systems.^6^, ^8^, ^9^, ^19^

**Figure 1:**
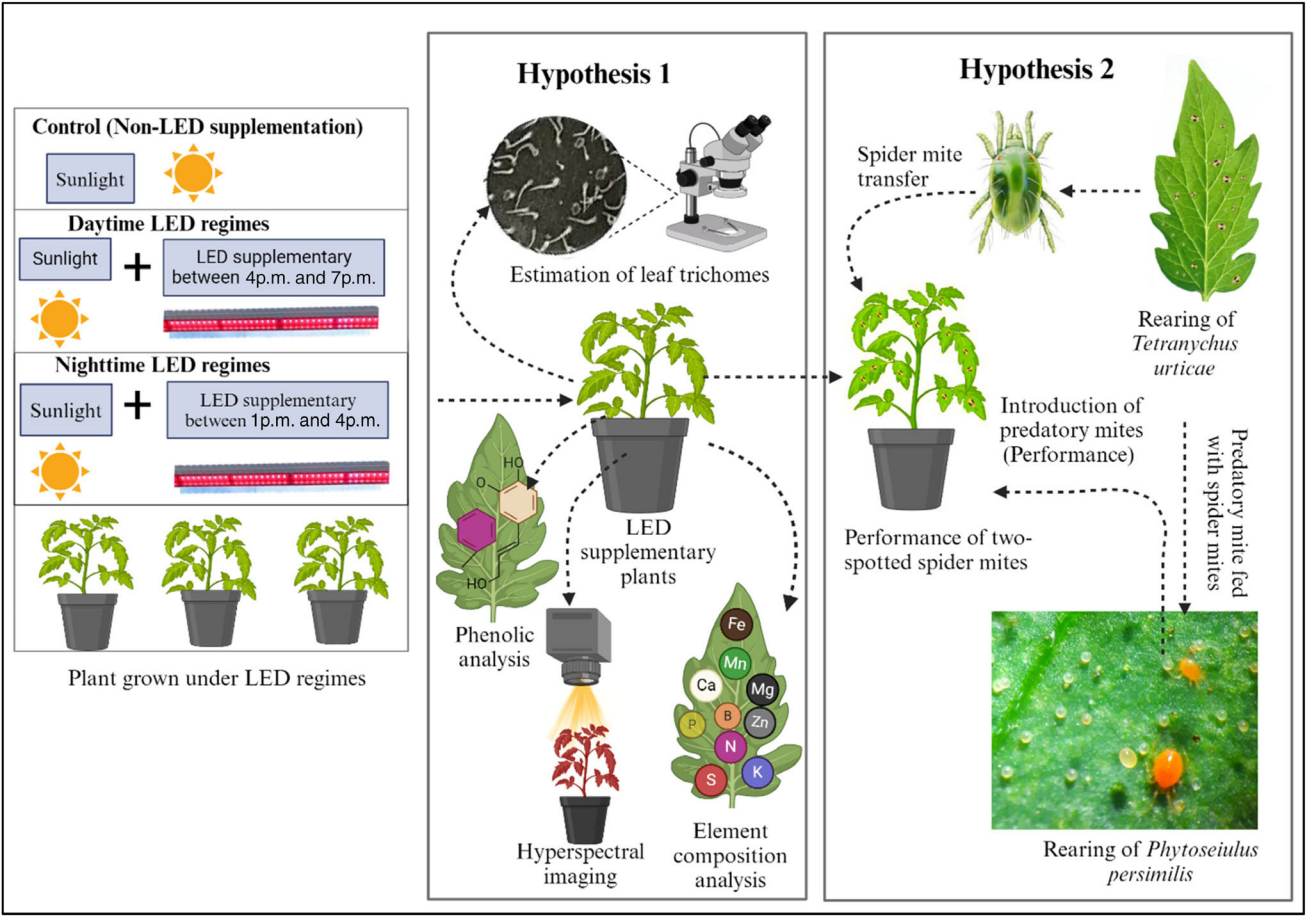
Experimental design.

Deployment of supplementing LEDs can also have indirect effects on arthropod pest populations and their natural enemies.^2^ Indirect effects can be manifested through changes in nutritional quality, secondary metabolites (including phenolic profiles), and physical traits (e.g., trichomes) of host plants.^4^, ^20^, ^21^, ^22^, ^23^, ^24^ Moreover, deployment of supplementing LEDs can reduce host plant suitability and alter pests' foraging behavior.^2^ For example, glandular trichomes are known to have deterrent effects on a number of important pest species,^25^, ^26^, ^27^ and density and toxicity of these trichomes have been found to increase when tomato plants are grown under supplemented red or blue LEDs.^24^ Figure 1 summarizes perceived direct and indirect effects of supplementary LEDs on plant growth, pest populations and their natural enemies.

Use of element composition, trichome density, and secondary metabolites as response variables in studies of indirect plant responses to supplementary LEDs and other treatments typically requires^28^, ^29^, ^30^ destructive sampling, are time‐consuming, and incur high costs.^28^, ^29^, ^30^ In contrast, optical sensing technologies offer a non‐destructive, rapid, and cost‐effective alternative.^31^, ^32^, ^33^ When conditions such as lighting, projection angle, and distance are controlled, reflectance features captured from plant leaves can provide reliable quantitative data related to plant physiological and biochemical traits.^34^, ^35^, ^36^ Reflectance indices, such as the Blue‐Green Index (BGI), Biochemical Reflectance Index (BRI), Anthocyanin Reflectance Index (ARI), Carotenoid Reflectance Index (CRI), and Normalized Difference Vegetation Index (NDVI), enable real‐time assessment of secondary metabolite profiles and photosynthetic efficiency in response to various treatments.^37^, ^38^ This approach reduces the subjectivity and representativeness concerns associated with destructive methods, allowing for a more comprehensive and precise analysis of plant responses. As shown in Fig. 1, leaf reflectance profiling could be used to non‐destructively detect and diagnose the effects of supplementary LED regimes, providing valuable insights into the physiological and biochemical responses of crop plants.

Most studies describing effects of supplementary LEDs on plant growth and/or on pest population dynamics utilize continuous LED regimes (applied throughout the entire daytime or nighttime period).^2^ However, recent research by Wang, *et al*.^21^ suggested that providing supplementary blue or red LED regimes at specific times can result in varying effects on plant quality traits. The authors found that morning LED supplementation showed a stronger influence on amino acids and carotenoids, while evening light supplementation for 3 h increased sugars, flavonoids, and aromatics in tomato fruits. These findings open exciting possibilities for optimization of both crop production and integrated pest management based on timed LED supplementation.

In this study, we conducted experiments with tomato plants (*Solanum lycopersicum*) to concurrently assess both direct and indirect effects of supplementary LED regimes on two‐spotted spider mites (TSSM) [*Tetranychus urticae* Koch (Acari: Tetranychidae)], and a species of predatory mites [*Phytoseiulus persimilis* Athias‐Henriot (Acari: Phytoseiidae)]. As outlined in Fig. 1, hypothesis 1 of this study is that deployment of supplementary LEDs during the day or at night has significant indirect effects on tomato plants, including reflectance indices, plant nutritional quality (element composition) and defensive traits (trichome density and phenolic contents). Hypothesis 2 of this study is that simultaneous direct and indirect effects of supplementary LEDs during the day or at night elicit significant variations in performance of both TSSM and their natural enemy. TSSM is a widespread agricultural pest, known to inflict considerable damage to various greenhouse and field crops due to their short life cycle and high reproductive capacity.^39^, ^40^ Traditional control methods of this pest rely heavily on applications of synthetic acaricides.^41^ According to Arthropod Pesticide Resistance Database (APRD, http://www.pesticideresistance.org/), TSSM have developed resistance to 96 active ingredients. Accordingly, use of natural enemies, such as commercially available predatory mite species including *Phytoseiulus persimilis* Athias‐Henriot (Acari: Phytoseiidae), is becoming widely adopted in greenhouse crop production systems and in other cropping systems.^39^, ^42^ The insights from this study will guide more effective implementation of LED regimes in integrated pest management (IPM) strategies, optimizing both plant growth and pest control in controlled environments.

## 2 MATERIALS AND METHODS

### 2.1 Plants and arthropods

We used seeds of the tomato variety BQ273 (*Solanum lycopersicum* L.), which represents significant economic importance to tomato processing growers in California. Tomato plants were grown in glasshouse facilities at the University of California, Davis. Initially, seeds were sown in tray cells containing a homogeneous mixture of pumice, sphagnum peat moss, sand, redwood sawdust, and 5.23 kg of dolomite per cubic meter of soil. The soil mix was autoclaved at 121 °C for an hour. Trays were placed in the glasshouse, and an automated sprinkling irrigation system (Mix Rite injector, model 2502) provided water and fertilizer four times a day at 7 am, 10 am, 2 pm, and 5 pm. The fertilizer solution had the following composition (values in ppm): N = 150, P = 50, K = 200, Ca = 175, Mg = 55, S = 120, Fe = 2.5, Cu = 0.02, B = 0.5, Mn = 0.5, Mo = 0.01, and Zn = 0.05. Four weeks after sowing, seedlings were individually transplanted to 1.5‐L pots 80% filled with a homogeneous mixture of soil, sand, and tanned bovine manure (1:1:1) autoclaved at 121 °C for 1 h. Tomato plants were then moved to compartments within the glasshouse exposed to natural light conditions (25.2 ± 1 °C, 77 ± 10% RH, and a 12:12 light–dark photoperiod) with supplementary LED regimes. Each compartment contained two rows with six plants per row, totaling 12 plants per supplementary light treatment. Two parallel full‐spectrum LED sources were installed in each compartment at 20 cm above a light table with experimental plants to provide supplementary light, with red, blue, and far‐red LEDs at rates of 16, 27, and 17 μmol m^−2^ s^−1^, respectively, throughout the experiment. The fertilizer was delivered to individual pots through drip irrigation four times a day as mentioned previously. Each irrigation session lasted for 1 min, resulting in a total daily volume of 140 mL (4 × 35 mL).

TSSM colony used in this study was reared on tomato plants for several generations in a greenhouse at the University of California, UC Davis, USA. A colony of predatory mites, *P. persimilis*, originally obtained from Koppert Biological Systems Inc., was established on TSSM infested tomato leaves using the methodology described by Savi, *et al* ^26^ Both colonies were maintained under the following conditions: 25.2 ± 1 °C, 77 ± 10% RH, and a L:D 12:12 photoperiod.

### 2.2 Light treatments

Supplementary LED regimes used in this study included red light (λmax = 667 nm), blue light (λmax = 462 nm), and far‐red light (λmax = 741 nm). LED regimes involved 3‐h exposure either during the day (5 p.m. to 8 p.m.) or at night (1 a.m. to 4 a.m.). To provide a baseline for comparison, control plants without any LED regime were also included. Thus, 12 tomato plants (replicates) were assigned to each of the following seven treatments: 1) Control (non‐LED supplementation), 2) Red‐Day, 3) Red‐Night, 4) Blue‐Day, 5) Blue‐Night, 6) Far‐red‐Day, and 7) Far‐red‐Night.

### 2.3 Element composition and trichomes assessment

Two weeks after light treatments, leaf samples were collected from the median third of the plant canopy for analysis of elemental composition (N, P, K, Ca, Mg, S, Fe, Cu, B, Mn, Zn and Na). Each sample consisted of 0.5 g of dried leaflet material obtained from three plants, resulting in four samples per treatment. Element composition analyses were performed by University of California Davis analytical laboratory, and standard methods for analyses can be found at https://anlab.ucdavis.edu. Regarding trichome assessment, six randomly selected plants with five leaflets each were taken from the middle third of the canopy, totaling 30 leaflets per treatment. Glandular and non‐glandular trichomes were counted in a 1 square mm leaflet area on both sides near the middle portion of the main vein following the methodology described by.^34^ The counting was performed using a 10× magnification microscope (Olympus SZ61) with a digital camera (Olympus DP27) and Cell Sens Entry software.

### 2.4 Leaf reflectance

Hyperspectral optical sensing data were obtained before experimental infestations with TSSM from six tomato plants for each of the seven light treatments (*n* = 42). This technology was utilized to non‐destructively and quantitatively characterize the effects of LED treatments on tomato plants. Optical sensing data were acquired using a push‐broom hyperspectral camera (PIKA L, www.resonon.com) following the methodology described previously.^35^, ^36^ We collected optical sensing data in 150 spectral bands ranging from 380 to 1015 nm (with a spectral resolution of 4.2 nm). The hyperspectral camera was mounted on a custom‐built robotic rail system, which moved approximately 1.5 m above the tomato plants. As a result, the spatial resolution of the optical sensing data was approximately 9 pixels/mm^−2^. The data were acquired around 4 PM within a dark room maintained at a temperature of 20–23 °C and a relative humidity of 50–75%. To provide an active light source, one row of 12 15 W 12 V halogen light bulbs was mounted on each side of the lens. A piece of white Teflon was used for white calibration, and both dark and white calibrations were recorded to obtain relative reflectance.

### 2.5 HPLC analysis of phenolic compounds and flavonoids

Two weeks after light treatments, leaf samples were collected from the median third of the plant canopy for analysis of two classes of phenolic compounds monolignols (ferulic esters) and flavonoids. A total of 0.5 g of dried leaflet material from different treatment plants was ground into a fine powder for HPLC analysis following methodology adapted from^43^ in the Kliebenstein Lab at the Department of Plant Sciences, UC Davis. Extraction of phenolic compounds and flavonoids was performed using 80% methanol, with the samples sonicated for 30 min and then centrifuged at 4000 rpm for 10 min. The resulting supernatant was filtered through a 0.45 μm syringe filter before injection into the HPLC system. HPLC analysis was carried out using an Agilent 2100 HPLC system (Agilent., Santa Clara, CA, USA), equipped with a UV–Vis Diode Array Detector (DAD). Separation was achieved using an Agilent RP‐C18 reverse‐phase chromatography column (250 mm length, 4.6 mm internal diameter, and particle size 5 μm). The mobile phase consisted of solvent A (water with 0.1% formic acid) and solvent B (acetonitrile with 0.1% formic acid). A gradient elution program was applied, starting at 5% solvent B and ramping up to 95% solvent B over a period of 30 min, followed by a re‐equilibration to initial conditions. The flow rate was maintained at 1.0 mL min^−1^, and the column temperature was set to 30 °C. Detection of phenolic compounds and flavonoids was performed at 280 nm, which is optimal for monitoring these compounds. Due to the lack of specific standards, the results were expressed in relative units (milli‐absorbance units, mAu) per 20 mg of tissue, allowing for comparisons across different treatments.

### 2.6 Performance experimental design

Two weeks after treatments, all experimental plants were infested with TSSM. Each plant was limited to six leaves, with five TSSM adult females placed on each leaf, totaling 30 mites per plant. TSSM mobile stages were counted at 1‐, 2‐, and 3‐weeks post‐infestation using a 10× magnifying glass. At week 3, six randomly selected plants per light treatment were destroyed to count TSSM eggs and mobile stages under a stereomicroscope. For the remaining plants, four adult *P. persimilis* predatory mites were introduced per plant to assess predator–prey interactions. The introduction of four predatory mites per plant was based on documented thresholds to ensure prey availability was not a limiting factor.^44^ One week later, all infested leaves were removed, and mobile and egg stages of both species were counted under stereomicroscope. Predation efficiency was determined using this formula: Predation efficiency = 100*(initial TSSM population ‐ final TSSM population) / initial TSSM population. TSSM populations on plants under different LED regimes were statistically similar, so egg counts from destroyed plants were assumed equivalent to those on remaining plants used for predatory mite release.

### 2.7 Statistical analyses

All data processing, analyses, and classifications were performed in R v3.6.1 (The R Foundation for Statistical Computing, Vienna, Austria). Effects of LED regimes and time (weeks) on the TSSM mobile population growth were analyzed using generalized linear mixed models (GLMM) from ‘lme4’ package for a repeated measures analysis. Data showed overdispersion relative to a Poisson distribution, so a negative binomial distribution was used for fitting. Explanatory variables included LED regimes and time (1, 2, and 3 weeks), with fixed factors for regimes and time and random effects for repeated measures within each plant. Significance of treatment, time effects, and their interactions were assessed using likelihood‐ratio tests (*P* < 0.05). Analysis of TSSM weekly data (eggs and mobile stages) and predatory mite numbers at week 4, was performed using a generalized linear model (GLM) with a quasi‐Poisson distribution. Analysis of predation efficiency was determined using GLM with a quasi‐Binomial distribution. Trichome density data were analyzed with a GLM and quasi‐Poisson distribution due to non‐normal distribution and overdispersion. Significant differences between treatments were followed by post‐hoc Tukey's tests (*P* < 0.05) using the ‘emmeans’ function in the ‘multcomp’ package.^45^ Data of element composition and phenolic contents (ferulic esters and flavonoids) were analyzed with ANOVA and Tukey HSD (*P* < 0.05) post‐hoc tests for significant mean comparisons. Principal Component Analysis (PCA) was conducted for significant data on leaf element composition and trichomes densities under different LED regimes, using a correlation matrix to explore relationships and patterns.

For the optical sensing data, we used a customized radiometric filter to ensure that only green pixels from each tomato plant were included in the statistical analyses. We collected hyperspectral profiles from a total of 7894 pixels (an average of 188 per tomato plant), which we used to classify treatments using support vector machine (svm) modeling. For the svm modeling, we used the library(e1071) with a radial kernel function and no specific hyperparameters (i.e., cost or gamma). To assess the classification performance, we calculated Kappa values^46^ and conducted 10‐fold cross‐validation.^47^, ^48^, ^49^ In k‐fold cross‐validation, training data sets are divided into ‘k’ equal portions with k = 10 in this study. The classification model is trained on ‘k‐1’ of these portions, while the remaining portion is used for validation. This process is repeated ‘k’ times, with each fold serving as validation, and results from ‘k’ tests are averaged to produce a single estimation of model performance. In a second analysis of optical sensing data, we generated average leaf reflectance for each tomato plant and conducted analysis of variance [library(multcomp)] to examine treatment effects in all 150 spectral bands. Leaf reflectance data can be used to calculate indices plant health and stress responses,^38^ including (Table 1): Anthocyanin Reflectance Index (ARI), Blue Green Pigment Index (BGI), Blue Red Pigment Index (BRI), Carotenoid Reflectance Index (CRI), Normalized Difference Vegetation Index (NDVI), and Photochemical Reflectance Index (PRI). ANOVA followed by Tukey HSD post‐hoc tests (*P* < 0.05) was conducted to examine treatment effects on leaf reflectance indices. For indices that did not meet assumptions of homoscedasticity and normality, the nonparametric Kruskal–Wallis's test was used to compare averages.

**Table 1:** Formulas for estimating hyperspectral vegetation indices

Anthocyanin Reflectance Index (ARI): $\mathrm{ARI}=\frac{1}{R550}-\frac{1}{R700}$ (1)
Blue Green Pigment Index (BGI): $\mathrm{BGI}=\frac{R450}{R550}$ (2)
Blue Red Pigment Index (BRI): $\mathrm{BRI}=\frac{R450}{R690}$ (3)
Carotenoid Reflectance Index (CRI): $\mathrm{CRI}=\frac{1}{R510}-\frac{1}{R550}$ (4)
Carotenoid Reflectance Index2 (CRI2): $\mathrm{CRI}2=\frac{1}{R510}-\frac{1}{R700}$ (5)
Normalized Difference Vegetation Index (NDVI): $\text{NDVI}=\frac{R800-R670}{R800+R670}$ (6)
Normalized Difference Vegetation Index (NDVI): $\text{NDVI}2=\frac{R750-R705}{R750+R705}$ (7)
Photochemical Reflectance Index (PRI): $\mathrm{PRI}=\frac{R531-R570}{R531+R570}$ (8)
Photochemical Reflectance Index2 (PRI2): $\mathrm{PRI}2=\frac{R570-R539}{R570+R539}$ (9)
Rx stands for reflectance at wavelength x nm.

## 3 RESULTS

### 3.1 Element composition

In tomato plants without TSSM infestation, we observed significant effects of LED regimes on average tomato plant content of the following elements: K, S, Mg, Zn, Mn, Cu (Table 2). Compared to the control, daytime regime with blue light significantly reduced Zn accumulation in tomato leaves. Nighttime regimes had varied effects: red light increased K accumulation, blue light increased Mg level, and far‐red light increased Mn content but decreased Zn level. Average tomato plant content of N, P, S, B, Ca and Fe were unaffected by LED regimes.

**Table 2:** Mineral contents in tomato plants under different LED supplementary regimes

Treatments	N	P	K	S	B	Ca	Mg	Zn	Mn	Fe	Cu
**Control**	4.8 ± 0. 2a	0.8 ± 0.1a	3.2 ± 0.2b	14 810 ± 2272.2a	39.1 ± 2.9a	3.1 ± 0.2a	1.0 ± 0.1b	30.4 ± 0.2a	55.2 ± 4.6bc	99.3 ± 5.3a	6.6 ± 0.6abc
**Red day**	4.6 ± 0.2a	0.7 ± 0.1a	3.1 ± 0.3b	13 250.0 ± 1909.2a	37.3 ± 3.0a	2.9 ± 0.4a	0.9 ± 0.1b	28.1 ± 1.8ab	58.4 ± 16.4abc	88.2 ± 0.4a	5.9 ± 0.3bc
**Red night**	4.8 ± 0.3a	0.8 ± 0. 1a	3.7 ± 0.2 a	13 836.7 ± 2896.6a	40.15 ± 0.1a	3.1 ± 0.1a	1.0 ± 0.1b	29.9 ± 0.8a	63.7 ± 2.6abc	101.7 ± 17.9a	6.9 ± 0.7abc
**Blue day**	4.7 ± 0.1a	0.8 ± 0.1a	3.3 ± 0.3ab	14 000.0 ± 1967.2a	39.0 ± 3.4a	3.0 ± 0.5a	0.9 ± 0.1b	26.7 ± 0.6b	46.4 ± 0.6c	85.5 ± 3.5a	5.8 ± 0.1c
**Blue night**	4.5 ± 0.2a	0.7 ± 0.03a	3.5 ± 0.1ab	15 240.0 ± 3035.38a	41.9 ± 0.9a	3.7 ± 0.1a	1.3 ± 0.1a	28.6 ± 0.1ab	55.3 ± 2.3bc	111.9 ± 3.7a	7.2 ± 0.2a
**Far‐red‐day**	4.7 ± 0.1a	0.7 ± 0.1a	3.1 ± 0.1b	12 442.5 ± 1853.5a	36.4 ± 4.2a	2.9 ± 0.6a	0.9 ± 0.1b	27.8 ± 1.6ab	72.4 ± 1.5ab	87.4 ± 1.2a	7.0 ± 0.7ab
**Far‐red‐night**	4.7 ± 0.1a	0.7 ± 0.0a	3.4 ± 0.2ab	14 582.5 ± 2201.2a	39.5 ± 1.6a	3.4 ± 0.2a	1.1 ± 0.1ab	26.3 ± 0.5b	76.4 ± 6.5a	96.3 ± 5.3a	7.2 ± 0.3ab
**F‐value**	0.36	2.48	0.04	3.82	1.29	2.08	5.35	7.79	6.50	3.07	5.13
**d.f**.	6,10	6,10	6,10	6,10	6,10	6,10	6,10	6,10	6,10	6,10	6,10
**P**	0.89	0.07	<0.01	0.01	0.34	0.11	0.01	0.002	<0.01	0.05	0.01

Means (±SE) within a column followed by different letters are significantly different (Tukey HSD test, *P* < 0.05).

### 3.2 Leaf reflectance indices

In comparison of average leaf reflectance indices acquired from control plants, we observed that (Table 3): (i) Nighttime regimes with red and blue LEDs significantly increased ARI, and CRI2. (ii) Both daytime and nighttime far‐red LED regimes significantly reduced ARI, and CRI2 levels. (iii) Nighttime far‐red LED regime significantly decreased BGI, BRI, and NDVI2 levels. No significant differences were observed between the control and other LED regimes for ARI, CRI2, and NDVI2, and PRI2 levels remained unaffected by any LED regimes.

**Table 3:** Estimation of Anthocyanin Reflectance Index (ARI), BGI (Blue Green Pigment Index), BRI (Blue Red Pigment Index), Carotenoid Reflectance Index (CRI), Photochemical Reflectance Index (PRI) and Normalized Difference Vegetation Index (NDVI) based on 45‐day‐old supplemented LED‐ tomato plants

Treatments	ARI^a^	BGI^b^	BRI^b^	CRI^a^	CRI2^b^	PRI^b^	NDVI^a^	NDVI2^a^
Control	0.0023b	0.067a	0.063a	0.012b	0.014b	0.057a	0.867a	0.56ab
Red day	0.0021bc	0.067a	0.063a	0.011bc	0.013bc	0.056a	0.865a	0.56ab
Red night	0.0025a	0.065a	0.061b	0.014a	0.016a	0.054a	0.871a	0.57a
Blue day	0.0021bc	0.066a	0.063a	0.011bc	0.013bc	0.053a	0.867a	0.56ab
Blue night	0.0025a	0.069a	0.063a	0.015a	0.017a	0.053a	0.868a	0.58a
Far‐red‐day	0.0019 cd	0.065a	0.061a	0.010c	0.012 cd	0.052a	0.865a	0.56b
Far‐red‐night	0.0018d	0.059b	0.059b	0.010c	0.011d	0.054a	0.865a	0.53c
F	26.25	‐	‐	11.45	‐	‐	5.37	17.59
ꭓ^2^	‐	28.91	22.31	‐	48.08	9.72	‐	‐
df	6,77	6	6	6,77	6	6	6,77	6,77
*P*‐value	<0.0001	<0.001	<0.001	<0.0001	<0.001	0.13	0.49	<0.0001

^a^ Values within a column followed by different letters are significantly different (Tukey HSD test, *P* < 0.05).

^b^ Values within a column followed by different letters are significantly different (Kruskal–Wallis's test, *P* < 0.05).

### 3.3 Trichome densities

LED regimes, during the daytime and nighttime, significantly reduced non‐glandular trichome densities (F = 42.86; df = 6161; *P* < 0.001) compared to control plants (Table 4). Glandular trichome densities were higher with red and blue LED regime at nighttime (F = 9.84; df = 6,61; *P* < 0.001), while no significant differences were observed between control and other LED regimes.

**Table 4:** Means (±SE) density of non‐glandular and glandular trichomes (no. trichomes/mm^2^) on both abaxial and adaxial surface of leaves of 45‐day‐old supplemented LED‐ tomato plants

Treatments	Non‐glandular trichomes (II + III + V)	Glandular trichomes (I + IV + VI + VII)
Control	17.54 ± 1.33a	2.0 ± 0.32c
Red day	6.83 ± 0.66b	3.25 ± 0.37bc
Red night	4.91 ± 0.53bc	6.0 ± 0.41a
Blue day	5.04 ± 0.54bc	3.04 ± 0.35bc
Blue night	4.33 ± 0.49c	3.75 ± 0.42b
Far‐red‐day	3.66 ± 0.44c	2.70 ± 0.33bc
Far‐red‐night	3.25 ± 0.41c	2.95 ± 0.35bc
F	42.29	9.81
d.f.	6161	6161
*P*. value	<0.001	<0.001

Means (±SE) within a column followed by different letters were significantly different (GLM with a quasi‐Poisson distribution, followed by *post hoc* Tukey's test with Šidák‐adjusted confidence intervals; *P* < 0.05).

### 3.4 Phenolic content and flavonoids

**Figure 2:**
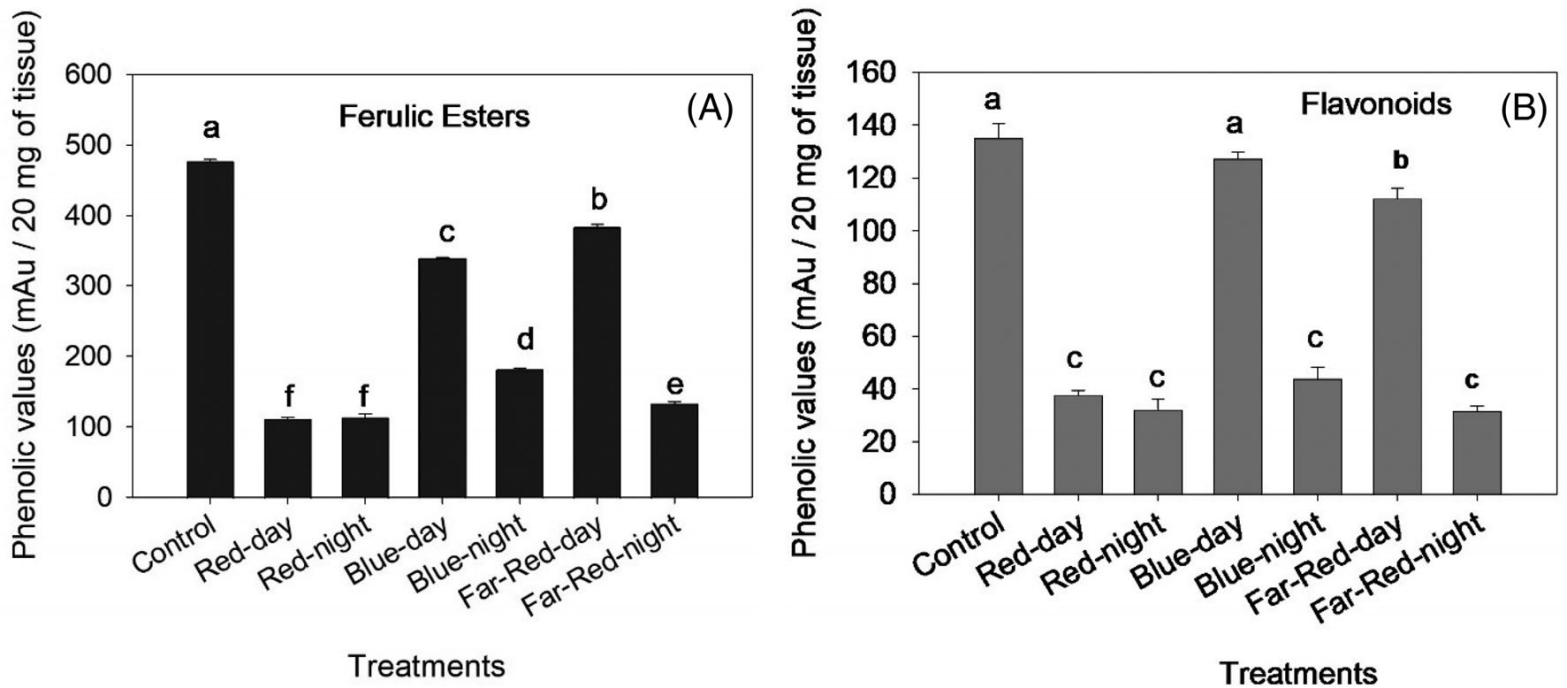
Effects of different LED regimes on the total phenolic content of ferulic esters and flavonoids in tomato leaf samples. Bars represent the mean phenolic values (mAU/20 mg of tissue) for each treatment. Different letters above each box indicate significant differences between treatments (Tukey HSD test, *P* < 0.05).

Daytime LED regime with red and all nighttime LED regimes showed significantly lower values for ferulic esters (F = 4585.5; df = 6, 7; *P* < 0.001) and flavonoids (F = 346.65; df = 6, 7; *P* < 0.001) compared to the control treatment and daytime LED regimes with blue and far‐red (Fig. 2).

### 3.5 Performance of TSSM and predatory mite

**Figure 3:**
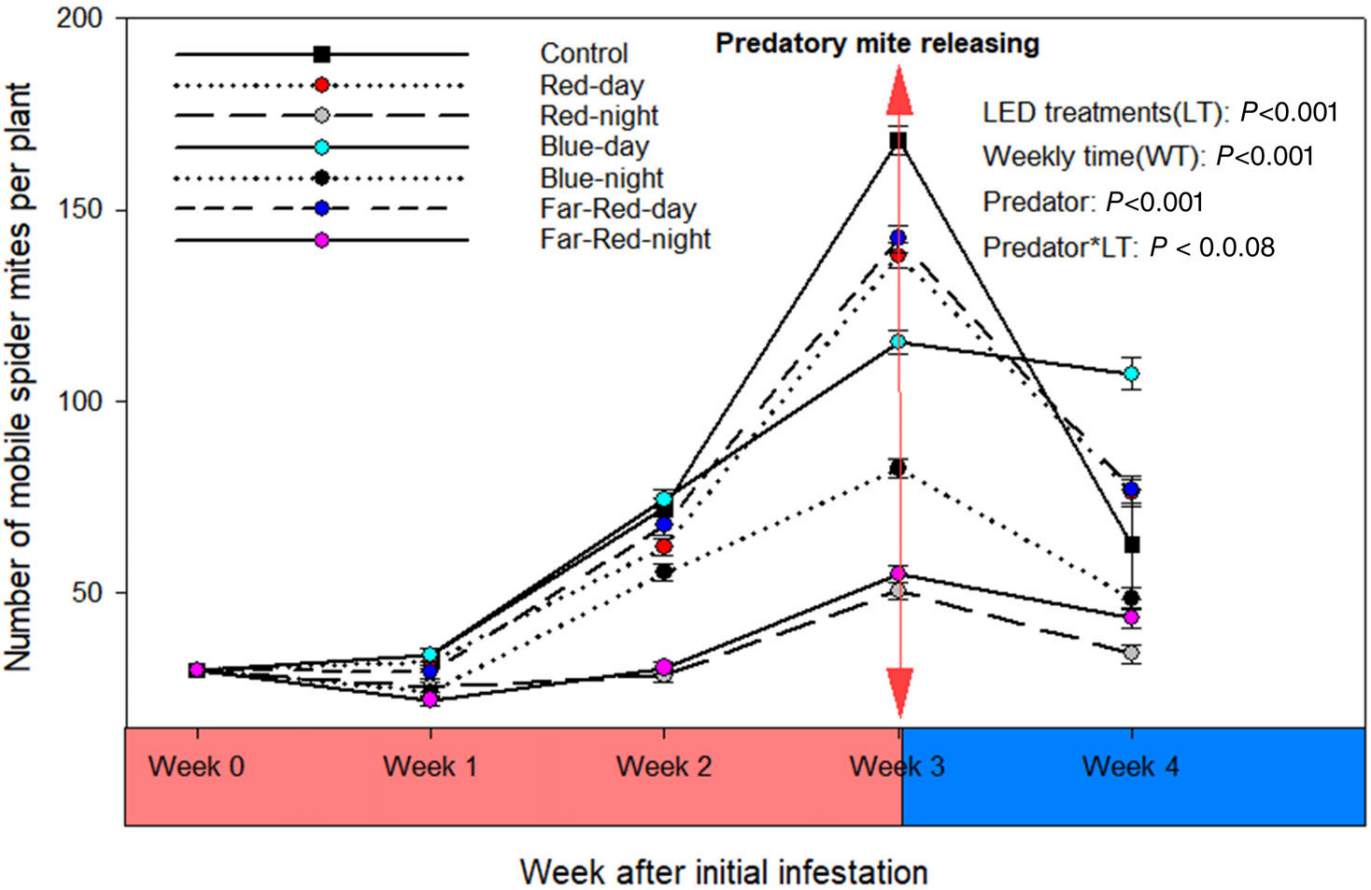
Average number (± SE) of mobile two‐spotted spider mites recorded over 4‐week after mite infestation on plant growth under daytime and nighttime exposure to different supplemental LEDs regimes (initial females infested/ plant = 30, number of replicates =12). Introduction of predatory at 3‐week after infestation (four females per plant, *n* = 6).

Population growth of mobile TSSM was significantly affected by LED regimes, presence of predatory mites, and LED regimes‐time interactions (GLMM χ^2^ = 74.55, df = 6; *P* < 0.001), but not by LED‐predatory mite interactions (GLMM χ^2^ = 8.26; df = 6; *P* = 0.21) (Fig. 3). One week after infestation, nighttime regimes with far‐red (22.2 ± 1.75 mites/plant), blue (24.4 ± 1.88), or red LED (25.5 ± 1.94) resulted in significant smaller TSSM population growth delays compared to the control (33.8 ± 2.39) (F = 4.68; df = 6, 77; *P* < 0.001). No significant differences were found between the control and daytime LED regimes (29.5–33.8 ± 2.15–2.39).

In the second week, nighttime red (28.6 ± 3.70) and far‐red (30.6 ± 3.94) LED regimes continued to show significant smaller mobile TSSM than the control (72.3 ± 8.86) and other LED regimes (55.4–72.3 ± 6.87–8.86) (F = 8.22; df = 6, 77; *P* < 0.001). After 3 weeks, red and far‐red LED regimes showed a three‐fold decrease, and blue LED treatment a two‐fold decrease, in mobile TSSM (50.7 ± 12.7 and 55.2 ± 13.2, respectively) compared to the control (168.2 ± 23.1) (F = 6.45; df = 6, 77; *P* < 0.001). TSSM egg numbers were also significantly lower with nighttime red (45.0 ± 16.5), blue (47.0 ± 16.9), and far‐red LED (60.5 ± 19.1) compared to the control (174.7 ± 32.5) (Fig. 3(B)) (F = 4.55; df = 6, 35; *P* < 0.001).

**Figure 4:**
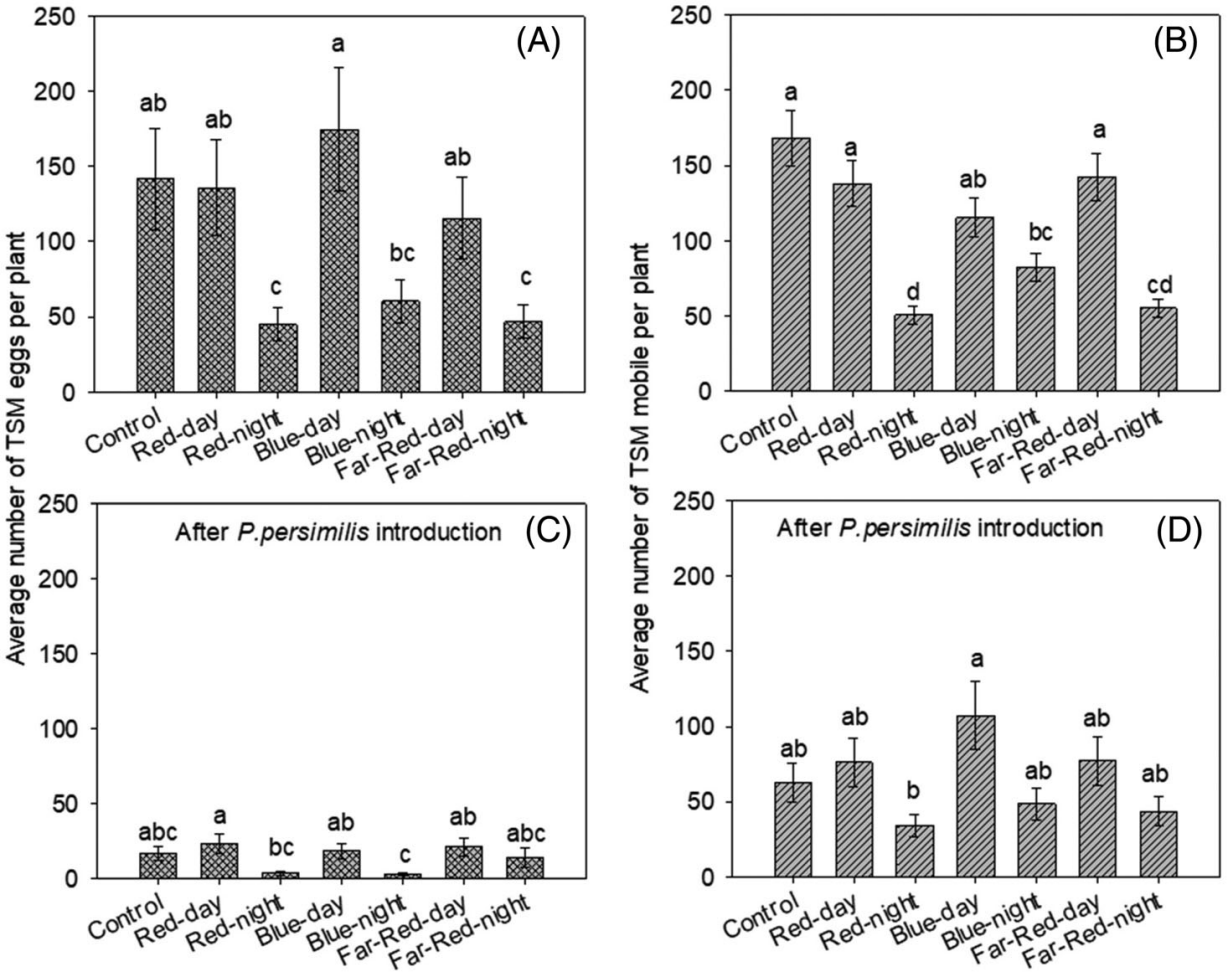
Average number (± SE) of mobile two‐spotted spider mites recorded 3‐week after initial mite infestation on plant growth under different supplemental lighting regimes (initial females infested/ plant = 30, number of replicates =12. Bars followed by the same letter for each assessment do not differ significantly (GLM with quasi‐Poisson distribution, followed by Šidák *post hoc* test; *P* < 0.05).

Four weeks post‐infestation, introducing predatory mite significantly reduced TSSM eggs (F = 5.052; df = 6, 35; *P* < 0.001) (Fig. 4(A), (C)) and mobile stages (F = 3.24; df = 6, 35; *P* = 0.01) (Fig. 4(B), (D)). There was no significant interaction between LED regimes and predatory presence in reducing mobile TSSM (χ^2^ = 0.85; df = 6, 28; *P* = 0.54) and TSSM eggs (F = 7.73; df = 6, 28; *P* = 0.86) (Figs 3 and 4(C), (D)). Predation efficiency on TSSM eggs (F = 0.73; df = 6, 35; *P* = 0.62), mobile mites, and combined stages (F = 7.73; df = 6, 28; *P* = 0.75) was consistent across all LED regimes, except for Blue LED regime, which showed reduced efficiency on mobile mites (F = 4.68; df = 6, 35; *P* < 0.001) (Table 5).

**Table 5:** Predation efficiency (±SE) of *Phytoseiulus persimilis* on eggs, mobile and on both TSSM egg and mobile stages of two spotted spider mites (TSSM) on supplemented LED‐ tomato plants

Treatments	Predation efficiency (%) on TSSM eggs	Predation efficiency (%) on mobile TSSM	Predation efficiency (%) on both TSSM egg and mobile stages
Control	88.1 ± 4.7a	65.6 ± 14.4a	75.0 ± 7.1a
Red day	82.7 ± 5.6a	52.8 ± 16.0a	66.3 ± 8.1a
Red night	92.2 ± 6.9a	33.5 ± 26.8ab	59.6 ± 14.9a
Blue day	89.5 ± 4.0a	25.9 ± 14.9b	57.1 ± 8.5a
Blue night	94.3 ± 5.8a	51.4 ± 20.4ab	63.5 ± 11.9a
Far‐red‐day	81.8 ± 6.2a	43.7 ± 17.3ab	61.0 ± 9.0a
Far‐red‐night	77.9 ± 9.1a	40.0 ± 23.5ab	56.2 ± 12.7a
F	0.73	4.68	7.73
d.f.	6,35	6,35	6,35
*P*. value	0.623	<0.001	0.75

Predation efficiency = (Initial TSSM population ‐ Final TSSM population) / Initial TSSM population.

Predation efficiency (±SE) within a column followed by different letters were significantly different (GLM with a quasi‐Binomial distribution, followed by *post hoc* Tukey's test with Šidák‐adjusted confidence intervals; *P* < 0.05).

**Figure 5:**
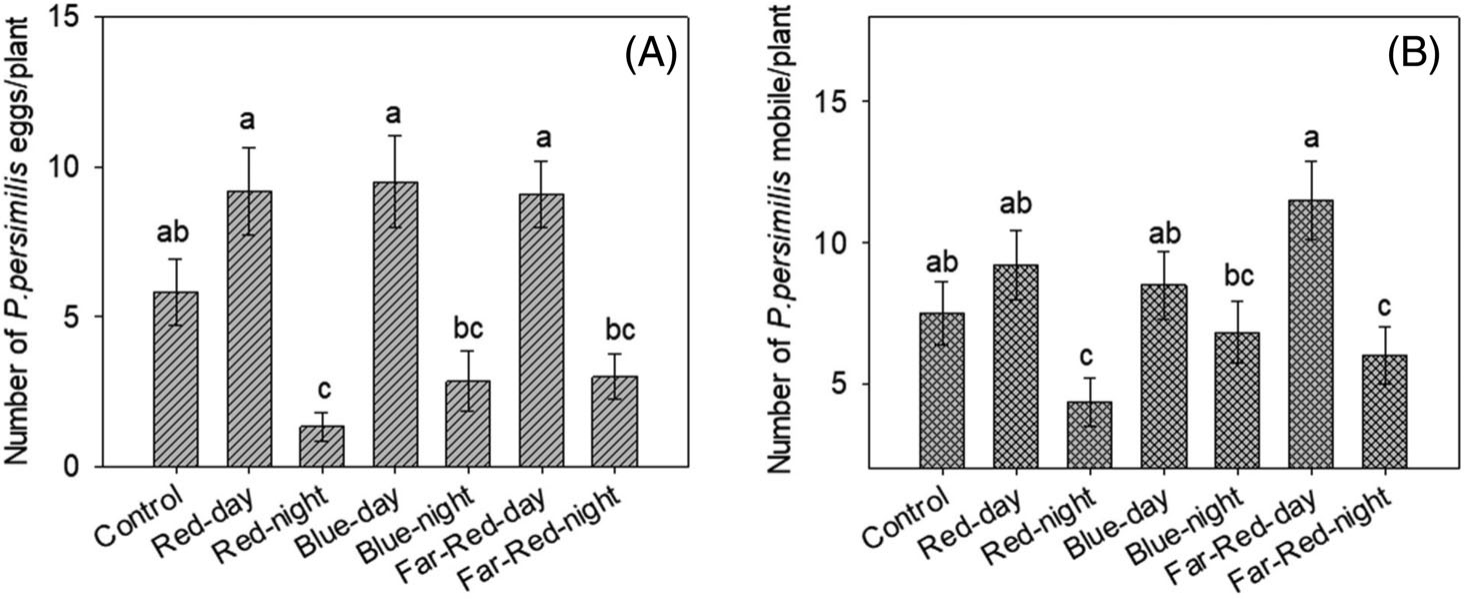
Average number (± SE) of *P. persimilis* eggs (A) and mobile stages (B) recorded 1‐week after on plant growth under different supplemental lighting regimes (initial females infested/ plant = 30; number of replicates = 6. Bars followed by the same letter for the number of *P. persimilis* eggs do not differ significantly. A. GLM with zero‐inflated negative binomial distribution, followed by *post hoc* Tukey's test with Šidák‐adjusted confidence intervals; *P* < 0.05. B. GLM with negative binomial distribution, followed by *post hoc* Tukey's test with Šidák‐adjusted confidence intervals; *P* < 0.05.

LED regimes had a significant effect on predatory mite egg numbers (F = 12.69, df = 6, 35; *P* < 0.001) (Fig. 5(A)) and mobile stages (F = 4.35, df = 6, 35; *P* < 0.001) (Fig. 5(B)). Initial TSSM population significantly affected predatory mite egg numbers (F = 8.17, df = 1; *P* = 0.007), but not mobile stages (F = 0.36, df = 1, 34; *P* = 0.55). The number of predatory mite eggs recorded in the control was comparable to those observed under all LED regimes, except for the nighttime red regime. Similarly, the number of predatory mite mobile stages in the control did not differ statistically from those observed under LED regimes, except for the nighttime red and far‐red regimes.

### 3.6 Principal component analysis (PCA)

**Figure 6:**
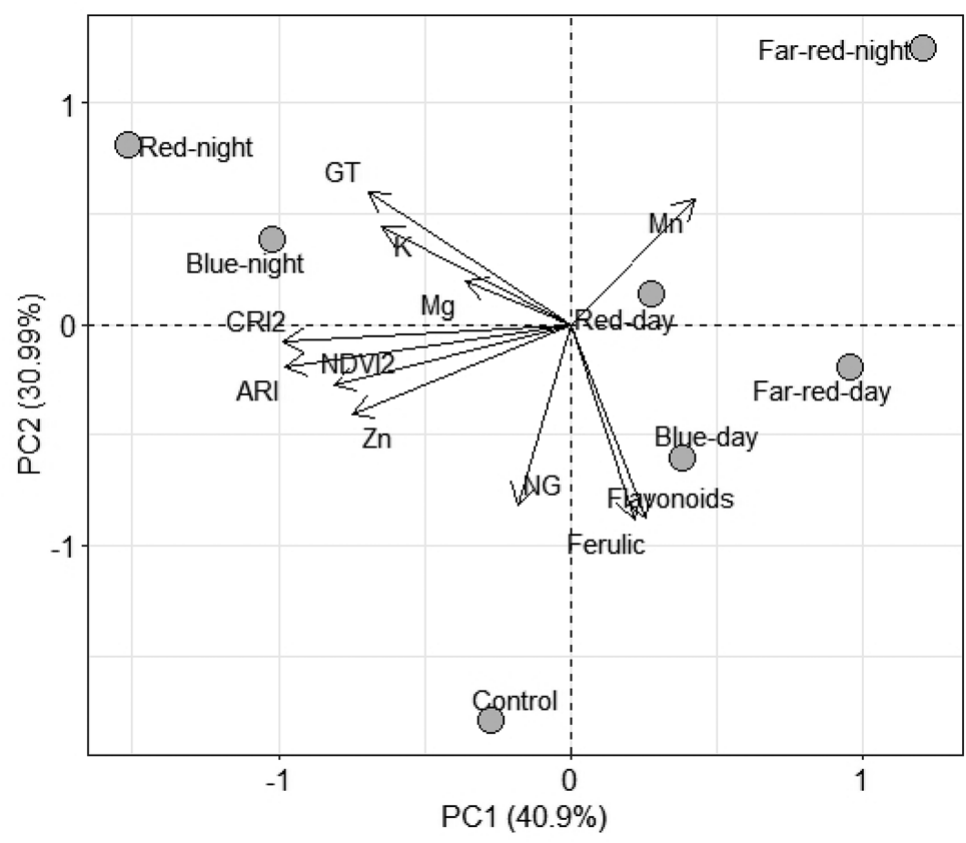
Principal component analysis (PCA) of nutrient content (Mg, K, Cu, and Mn), leaf reflectance indices (ARI‐Anthocyanin Reflectance Index, NDVI2‐Normalized Difference Vegetation Index), defensive traits (GT‐Glandular trichomes and NGT‐ Non‐glandular trichomes, ferulic esters, and flavonoids) from tomato control group and tomato plants supplemented with LEDs regimes.

PCA was performed to identify patterns in plant traits that might explain variations in both TSSM and predatory mite populations across different LED regimes (Fig. 6). The first two principal components, PC1 (40.9%) and PC2 (30.99%), together account for 71.99% of the total variation, capturing the main differences among the LED treatments. Daytime blue LED‐supplemented plants are positioned to the right along PC1, indicating strong associations with ferulic esters, and flavonoids. In contrast, red and blue nighttime LED‐supplemented plants are located on the left side of the plot, showing high associations with glandular trichomes (GT), Mg, K, and ARI. The control treatment has high positive loadings for non‐glandular trichomes (NG) on PC2. Far‐red nighttime LED‐supplemented plants showed a strong positive loading for Mn on PC2, highlighting its distinct impact on this nutrient.

## 4 DISCUSSION

Light‐emitting diode (LED) based light sources, which can selectively and quantitatively provide different spectra, have been frequently applied to manipulate plant growth and pest management strategies in greenhouse facilities with most existing studies focused on continuous LED regimes.^50^, ^51^ Here, we investigated hypothesis that providing tomato plants with timed LED regimes (daily 3‐h doses of red, blue, or far‐red LED) during the day or at night may affect tomato plant traits (leaf reflectance indices, element composition, and phenolic profile), performance of TSSM, and a predatory mite species *Phytoseiulus persimilis*. Our results indicate that daytime regimes resulted in a significant reduction of non‐glandular trichomes in comparison with ambient sunlight control. Additionally, daytime blue LED regime significantly reduced Zn leaf nutrient content. Conversely, the effects of nighttime regimes varied significantly depending on the LED spectrum. For instance, red LED increased K levels, blue LED enhanced Mg level, and far‐red light increased Mn and Cu levels but decreased Zn content. Nighttime LED regimes significantly increased leaf glandular trichome densities and reduced phenolic contents including ferulic esters, flavonoids compared to daytime LED regimes and control. Leaf reflectance results showed a significant increase in ARI and CRI2 levels under nighttime red and blue LED regimes, and a decrease under far‐red LED regime. Additionally, nighttime far‐red LED reduced BGI, BRI, and NDVI2 levels. Performance bioassays showed significantly lower population of TSSM populations on nighttime LED supplemented‐ plants than on daytime regimes and control plants. LED regimes did not interfere with the predatory mite ability to prey on both egg and mobile TSSM populations, except for the daytime blue LED, which reduced predation on mobile TSSM. Additionally, LED regimes had no measurable impact on predatory mite populations, except under nighttime red and far‐red LED regimes. These results support our hypothesis that the response strength to LED regimes in plant characteristics and their interaction with higher trophic levels depend on the timing of supplementation.

**Plant responses to LED regimes**. Daytime regimes with red, blue, or far‐red LEDs had minimal effects on nutrient accumulation in tomato leaves, with the only apparent effect being a reduction in Zn accumulation under blue LED treatment. Additionally, all wavelengths tested significantly reduced non‐glandular trichomes. These findings differ from what is commonly observed under continuous LEDS, where blue light enhances nutrient uptake, and red light promotes biomass production. For instance, Kobori, *et al*.^52^ reported on Impatiens plants, that 12‐h continuous daily supplementation of red and blue dichromatic LEDs (6 am to 6 pm) led to a notable increase of 41.0% trichomes number on leaves. Similarly, Kalaitzoglou, *et al*.,^53^ reported that a continuous far‐red light regime in tomato plants led to growth and morphological changes but did not fully compensate for the absence of natural sunlight, reinforcing the concept that plants prioritize sunlight over artificial light. In contrast, nighttime regimes had significant effects on tomato plant physiology, as indicated by variations in reflectance indices. Red and blue nighttime LED regimes consistently led to higher Carotenoid Reflectance Indices (CRI), suggesting increased carotenoid production, which is vital for photosynthetic efficiency.^54^ These findings coincided with elevated K levels observed under nighttime red LED regime and increased Mg levels under nighttime blue LED regime observed in our study, both of which are known to enhance photosynthetic efficiency and are critical for chlorophyll production.^55^, ^56^ On the other hand, far‐red LED regimes, while increasing Mn and Cu levels, showed a decrease in Zn content, which might contribute to the lower CRI2, and other indices including BGI, BRI, and NDVI2 values reflecting suboptimal conditions for pigment accumulation and overall plant health. This suggests that the nutrient imbalances induced by far‐red light could be linked to the reduced pigment accumulation observed under this treatment. Moreover, nighttime LED regimes significantly alter plant defensive traits by increasing glandular trichome densities (except far‐red night treatment) and selectively modulating phenolic compound profiles. For instance, total phenolic content, including compounds such as ferulic esters and flavonoids, was significantly reduced. Conversely, anthocyanins—a specific subclass of phenolic compounds known to support stress responses—showed a notable increase, as reflected in the Anthocyanin Reflectance Index (ARI).^37^, ^38^, ^57^ This selective response suggests that nighttime LEDs trigger a shift in metabolic resource allocation, favoring anthocyanin biosynthesis over other phenolic pathways. These results are not surprising as the circadian timing of LED supplementation plays a crucial role in modulating nutrient uptake and secondary metabolite production. Nighttime LED regimes might act as a stress signal, disturbing the plant's circadian clock and altering growth patterns and resource allocation,^58^, ^59^ as indicated by significant changes in plant nutrient content and an increase in glandular trichomes and anthocyanin content. These observations align with findings by Kondo, *et al*.^57^ who also reported increasing anthocyanin in grape berries under blue LED irradiation at night. Wang, *et al*.^21^ also found that nighttime supplemental LED lighting significantly impacted the nutrient content and secondary metabolites in tomato fruits, with red LED exposure increasing K levels, blue LED increasing Mg level, and far‐red LED light affecting the levels of Mn, Cu, and Zn. Similarly, Ueda, *et al*.^60^ reported that supplementing monochromatic blue, red, or far‐red light (16 h light/8 h dark) to a white LED regime (6 h) for a 2‐week period in Japanese Mint did not increase glandular trichome densities. In fact, blue LED regime even suppressed the formation of glandular trichomes. Although not directly investigated in this study, Paponov, *et al*.^61^ also demonstrated that nighttime regime inter LEDs (80% red, 20% blue; 70 W m^−2^; light period 04:00 a.m.–10:00 p.m.) elicited more efficient nutrient uptake and a higher concentration of certain phytohormones, such as jasmonate, which play a role in plant defense mechanisms. Similarly, Tewolde, *et al*.^58^ found that nighttime supplemental LED inter‐lighting from 10:00 p.m. to 10:00 a.m. not only increased the photosynthetic capacity and yield of tomato plants but also enhanced the total soluble solids and ascorbic acid content in tomato fruits. These results highlight the potential of nighttime LED supplementation in boosting plant growth and defenses and crop yield underscoring the need to better understand the timing of LED lighting in horticulture.

**LED supplementation plants with TSSM and P. persimilis interactions**. Nighttime LED regimes elicited a significant reduction in TSSM and predatory mite populations under red and far‐red nighttime LED regimes. These results are not surprising, possibly due to both plant‐induced defensive responses (indirect effects)^5^, ^62^ and disruption of TSSM circadian rhythms (direct effects).^63^ PCA results associated red and blue nighttime LEDs with increased glandular trichomes, Mg, K, and anthocyanin levels in tomato plants. The increase in glandular trichomes known to contain acyl sugars, methyl ketones, and sesquiterpenes, may have contributed deterred reduction of TSSM populations.^26^, ^64^ Additionally, improvements in K and Mg levels observed under red and blue nighttime LEDs may have enhanced plant resistance to TSSM, as K deficiency can limit plant growth, metabolism, and defense responses, thus benefiting arthropod herbivores.^65^, ^66^ Anthocyanins, widely recognized for their antioxidant properties and role in modulating secondary metabolic pathways,^67^, ^68^ can also deter herbivory and reduce pest fitness. Thus, the elevated anthocyanin levels observed in LED‐supplemented plants likely created an unfavorable environment for TSSM, further contributing to their population reduction. Moreover, since arthropod behaviors and physiological functions are generally aligned with natural light–dark cycles,^69^, ^70^ long term nighttime LED regimes supplementation could disrupt TSSM's internal biological rhythms, impacting their reproductive and developmental mechanisms,^63^ as seen in this study. However, predation efficiency under nighttime LED regimes was not observed to be affected on TSSM populations, suggesting that the changes in plant traits under nighttime LED regimes did not negatively impact the predator mite’ ability to control TSSM populations. These findings align with studies by Bennie, *et al*.^71^ who reported that nighttime lighting supplementary reduce pea aphid *Aphis fabae* and *Acyrthosiphon pisum* populations on flower head density without affecting predators or predator–prey interactions. However, Sanders, *et al*.^72^ found a significant adverse effect on parasitoid populations, driven by bottom‐up effects, including reduced pear aphid populations and decreased bean plant biomass under artificial nighttime lighting. Although not directly examined in this study, LED regimes may have elicited positive phototactic behavior in predatory mites, enhancing their hunting and feeding efficiency. Further studies are required to confirm this effect.

Daytime LED regimes by contrast showed a non‐significant increase in TSSM populations and no impact on predatory mite populations. This suggests that these LED regimes do not strongly influence TSSM or predatory mite dynamics although they seem to have a strong association with ferulic esters, and flavonoids—traits in PCA analysis. These results imply that daytime LEDs are less disruptive, offering a growth‐promoting option without compromising the ecological balance of TSSM and predatory mite interactions. These results corroborated findings by Dieleman, *et al*.^62^ who reported that providing far‐red with blue LED enhanced eggplant growth without affecting interactions between Western flower thrips (WFT), *Frankliniella occidentali*s Pergande (Thysanoptera: Thripidae), and predatory mites (*Amblyseius swirskii*). Similarly, Fraser, *et al*.^5^ also observed that red and blue supplemental LEDs improved plant growth without diminishing the effectiveness of the parasitoid wasp *Aphidius matricariae* (Hymenoptera: Braconidae) on *Myzus persicae* (Hemiptera: Aphididae) in greenhouse settings. However, Meijer, *et al*.^4^ who reported that supplemental far‐red LED during the daytime (9–10 a.m.) can increase *P. persimilis* populations due to consistent TSSM availability. The reduced predation efficiency observed on mobile TSSM stages under blue LED regimes is likely attributed to behavioral disruptions caused by the blue LED,^73^ rather than plant‐mediated changes. However, further research is required to fully elucidate these behavioral impacts and their implications for pest management strategies.

In conclusion, our study demonstrated that nighttime LED regimes reduce TSSM and *P. persimilis* populations in tomato plants by enhancing glandular trichomes, anthocyanin levels, and nutrient profiles. In contrast, daytime LED regimes had minimal effects on TSSM and predator mite populations, preserving ecological stability while supporting plant growth. These findings highlight the importance of optimizing LED timing when designing IPM strategies that promote both plant growth and effective biological control in controlled environments. Future studies should investigate how different LED spectra, when applied at various times, affect a broader range of pests and beneficial organisms beyond TSSM and *P. persimilis*. Additionally, it is crucial to assess the impact of LED timing on crop yield, even though existing studies have shown yield increases under both daytime and nighttime LEDs.^58^, ^61^ This comprehensive approach will help to carefully select LED timing and spectrum, essential for optimizing both crop productivity and IPM strategies in controlled environments.

## AUTHOR CONTRIBUTIONS

Conceptualization was performed by PJS, and data collection was conducted by AM, DK, MH, SH and PJS. Data analysis and draft writing was by PJS and CN. All authors reviewed the manuscript.

## CONFLICT OF INTEREST

The authors have stated no conflicts of interest.

## Data Availability

The data that support the findings of this study are available on request from the corresponding author. The data are not publicly available due to privacy or ethical restrictions.
